# Thinking clearly about climate change and mental health

**DOI:** 10.1177/10398562231172398

**Published:** 2023-04-26

**Authors:** Andrew James Amos

**Affiliations:** Division of Tropical Health and Medicine, College of Medicine and Dentistry, 104397 James Cook University, Townsville, Australia

**Keywords:** Climate change, suicide, public policy, administrative psychiatry

## Abstract

**Objective:**

To examine the quality and strength of evidence for an association between temperature increases caused by climate change and suicide used in policy documents to advocate for radical changes to healthcare systems in pursuit of decarbonisation.

**Method:**

The designs of articles collected in a systematic review which concluded that there was an association between climate change and increased rates of suicide were analysed for their capacity to support this conclusion. Complete US data covering temperatures and suicide rates between 1968 and 2004 was aggregated and analysed using linear regression to evaluate evidence for an association between temperature and suicide.

**Results:**

None of the articles collected in the review has a design capable of investigating whether there is an association between temperature increases caused by climate change and rates of suicide. At the national level increased annual US temperatures were associated with a decrease in the rate of suicide, and at the state level it was common for high average temperature states to have low rates of suicide and vice versa.

**Conclusions:**

Policy recommendations for radical changes in healthcare services have been based on misrepresented evidence. Policy makers should beware of recommendations that ignore scientific evidence to pursue faith-based goals.

According to a MJA editorial, medical colleges should champion climate change action, including decarbonising colleges, hospitals, and health systems.^1^ They note this conflicts with the common-sense position of many health stakeholders that while ‘climate change mitigation in general was considered…important… [t]hey saw providing best possible medical care to be the top priority in hospitals and were often concerned that patients’ health could be jeopardised by climate change mitigation measures’.^2^

The RANZCP has also recommended systemic changes with unknown impacts on patient health^3,4^ based on a UK RCPsych position statement.^5^ Despite identifying ‘few published studies at a system- or country-level’ a review of the impact of climate change on health by the RACP endorsed by the RANZCP recommended all Australian governments and healthcare systems ‘[c]ommit to delivering net zero healthcare by 2040’.^4^

The strongest claims linking climate change and negative mental health outcomes have been made for suicide.^6^ The RANZCP asserts an association between suicide and climate change, referring to one study^7^ and the RCPsych’s statement.^5^ The latter bases its claim on a systematic review which concludes that the observational evidence for a causal relationship is weak.^6^ The realities of suicide and climate change present significant barriers to the randomisation, blinding, and other controls used in experimental research to support causal inferences. As progress is therefore likely to continue to rely upon observational evidence, there will need to be a systematic approach to evaluating evidence that balances the need for action with evidentiary limitations.^7^

The common-sense position noted above suggests that health systems should prioritise health care over climate change action until strong evidence for change is available. This research examined the strength of the evidence adduced to support the claim that climate change increases suicide, via a critical summary of the systematic review cited by the RCPsych,^6^ and statistical analysis of data from the empirical paper cited by the RANZCP position paper.^8^

## Methods

The research collected in the systematic review by Thompson et al.^6^ was evaluated for its ability to test the hypothesis that increased temperature as a result of climate change increases the rate of suicide.

Burke et al.^8^ used data about suicide and temperature across the US and Mexico between 1968 and 2004^9^ to look for an association between temperature and suicide at the level of individual suburbs by calendar month. They did not analyse the relationship between temperature and suicide at the aggregate level by year or state. As climate change by definition refers to global changes over long periods of time rather than suburb level variations measured in months, the US dataset was aggregated and reanalysed at the level of states and years rather than at the level of suburbs and months.

Burke et al.^8^ fit a linear model with multiple group effects to project out the effects of year and state

$$y_{ismt}=f\left(T_{ismt}\right)+\gamma P_{ismt}+\mu_{im}+\delta_{st}+\varepsilon_{is}$$

• *i*-suburbs
• *s*-states
• *m*-months
• *t*-year
• $y_{ismt}$ -monthly suicide rate,
• $f\left(T_{ismt}\right)$ -temperature response function
• $P_{ismt}$ -monthly precipitation
• $\mu_{im}$ -suburb-by-month effects
• $\delta_{st}$ -state-by year effects

The new analyses aggregated suicide and temperature by state and year, weighted for population, and linear regressions were estimated for suicide rate and average temperature by year

$$y_{t}=\alpha T_{t}+\varepsilon_{t}$$

and for suicide rate and average temperature by state

$$y_{s}=\alpha T_{s}+\varepsilon_{s}$$

## Results

Table 1 summarises the articles reviewed by Burke et al.^8^ and analyses their ability to test whether temperature increases due to climate change influence suicide rates.

**Table 1.** Summary of articles cited as authority for a relationship between climate change and suicide

Article	Study designed to examine effect of climate change on suicide	Analysis
Ajdacic-Gross et al. (2007)	No	Concluded that there was an association between higher temperatures and suicide, but the main effect was that higher temperatures in winter allowed better access to outdoor means (jumping from heights/in front of trains)
	Designed to examine hypothesis that suicide related to seasons (seasonality hypothesis)	
	Examined all registered suicides in Switzerland between 1877 and 2000 (*n* = 128,322)	
Deisenhammer et al. (2003)	No	Only significant correlation was between temperature and suicide; on average, a 10% increase in temp associated with a 12% increase in risk of suicide (T_mean_RR = 1.13; *p* = 0.012)
	Designed to examine cross-sectional association of meteorological factors including daily temperature with suicide for all suicide deaths in Tyrol between 1995 and 2000 (*n* = 702)	
Dixon et al. (2007)	No	Found no correlation between suicide and temperature (*R*^2^ = 0.006; *p* < 0.05; statistically but not practically different from zero)
	Designed to examine correlation between monthly suicide and temperature in five US counties 1991–2001 (*n* = 3355)	
Dixon et al. (2014)	No	Found ‘warm’ weeks (32°C) were associated with higher suicide risk in Jul, Aug, and Sep (RR 2x compared with median weeks - 22°C)
	Designed to test whether weekly suicides were associated with ‘warm’ weeks in two different climates between 1986–2009 (Canada) and 1980–2006 (Mississippi) (*n* = 3355)	
Fernandez-Arteaga et al. (2016)	No	Found an association between days with no rain and temperature between 30°C and 40°C and death by hanging (standard statistics not reported)
	Designed to test for an association between daily completed suicide and temperature in Mexican population between 2005–2012 (*n* = 1357)	
Gaxiola-Robles et al. (2013)	No	Reported correlation between hot season and suicide (*R*^2^ = 0.68; *p* < 0.01)
	Designed to test for a correlation between seasonal temperature and suicide in Mexico 1985–2008(*n* = 582)	
Grjibovski et al. (2013)	No	Reported RR of suicide per 1°C = 1.37 (T_mean_)
	Designed to test for an association between daily temperature and suicide in Kazakhstan 2005–2010(*n* = 685)	
Hiltunen et al. (2014)	No	Inconsistent results - ‘there was diversity in our results, and no single explanatory variable was associated uniformly with suicide rates of men and women in the three study areas’.
	Designed to test for an association between daily temperature and suicide rates in Finland 1974–2010 (*n* = 10,802)	
Kim et al. (2016)	No	Found a range of associations between temperature and suicide across countries - country-level estimates 7.8% increase in suicide (95% CI: 5–10.8%) for a 2.3°C increase in temp in Taiwan; 6.8% (5.4–8.2%) for a 4.7°C increase in Korea; 4.5% (3.3–5.5%) for a 4.2°C increase in Japan
	Designed to test for an association between daily temperature and suicide in Korea (1992–2010), Japan (1972–2010), and Taiwan (1994–2007)(*n* = not reported)	
Kim et al. (2011)	No	Reported a 1.4% increase in daily suicide risk for every 1°C increase in temperature
	Designed to test for an association between daily temperature and suicide in Korea 2001–2005(*n* = 49,451)	
Lee et al. (2006)	No	Reported a correlation of 0.376 (*p* < 0.0001) between temperature and suicide
	Designed to test for an association between monthly temperature and suicide in Taiwan 1997–2003(*n* = 18,083)	
Likhvar et al. (2011)	No	Reported seasonal effects with suicide peaks in Spring and Autumn, and weekly effects with peaks on Mondays and Tuesdays; statistics reported at city-level
	Designed to test for an association between daily temperature and suicide in Japan 1972–1995(*n* = 501,950)	
Lin et al. (2008)	No	Reported correlation between temperature and violent means of suicide (r = 0.2; *p* < 0.001)
	Designed to test for an association between seasons and suicide in Taiwan 1997–2003 (*n* = 18,130)	
Maes et al. (1994)	No	Reported correlation between violent suicide and temp (r = 0.32; *p* < 0.0001)
	Designed to test for correlation between weekly temperature and suicide in Belgium 1979–1987(*n* = not reported)	
Marion et al. (1999)	No	Reported RR = 1.16 for 2.5°C change in average daily temperature (*p* < 0.05)
	Designed to test for the effect of monthly temperatures on suicide in British Columbia, Canada 1981–1991 (*n* = 758)	
Page et al. (2007)	No	Reported 3.8% increase in total suicides for each 1°C increase in temperature
	Designed to test the association between daily temperature and suicide in England and Wales 1993–2003 (*n* = 53,623)	Reported 41.5% increase in suicide deaths associated with a heatwave in 1995 relative to 10.8% increase in all-cause mortality; and no increase in suicide deaths associated with a 2003 heatwave
Qi et al. (2014)	No	Reported a 1°C increase in yearly mean temperature associated with 2.27% increase in suicide over 1996–2000 and 2.34% increase over 2001–2005
	Bayesian spatial analysis tested for association between monthly temperatures and suicides in Australia 1986–2005 (*n* = 45,293)	

Table 2 summarises the regressions estimating relationships between temperature, location, time, and suicide rate using aggregated and disaggregated data.

**Table 2.** Linear regressions estimating relationships between temperature and suicide

Model	Source	Coefficient	SE	t	*p*
Burke et al	Monthly temp	0.0067	0.00074	9.051	<0.001
	Monthly rain	−0.000035	0.000024	−1.472	0.141
	Adjusted *R*^2^ (full): 0.1358		Adjusted *R*^2^ (projected): −0.04797		—
Aggregate-year	Annual temp	−0.077	0.02254	−3.417	<0.001
	Adjusted *R*^2^: 0.229		—	—	—
Aggregate-state	Ave state temp	0.0336	0.00817	4.112	<0.001
	Adjusted *R*^2^: 0.249		—	—	—

**Figure 1.**
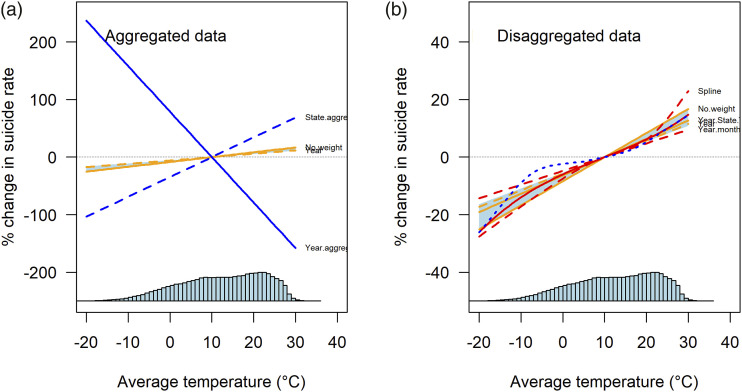
Estimated relationships between temperature and suicide (US).

**Figure 2.**
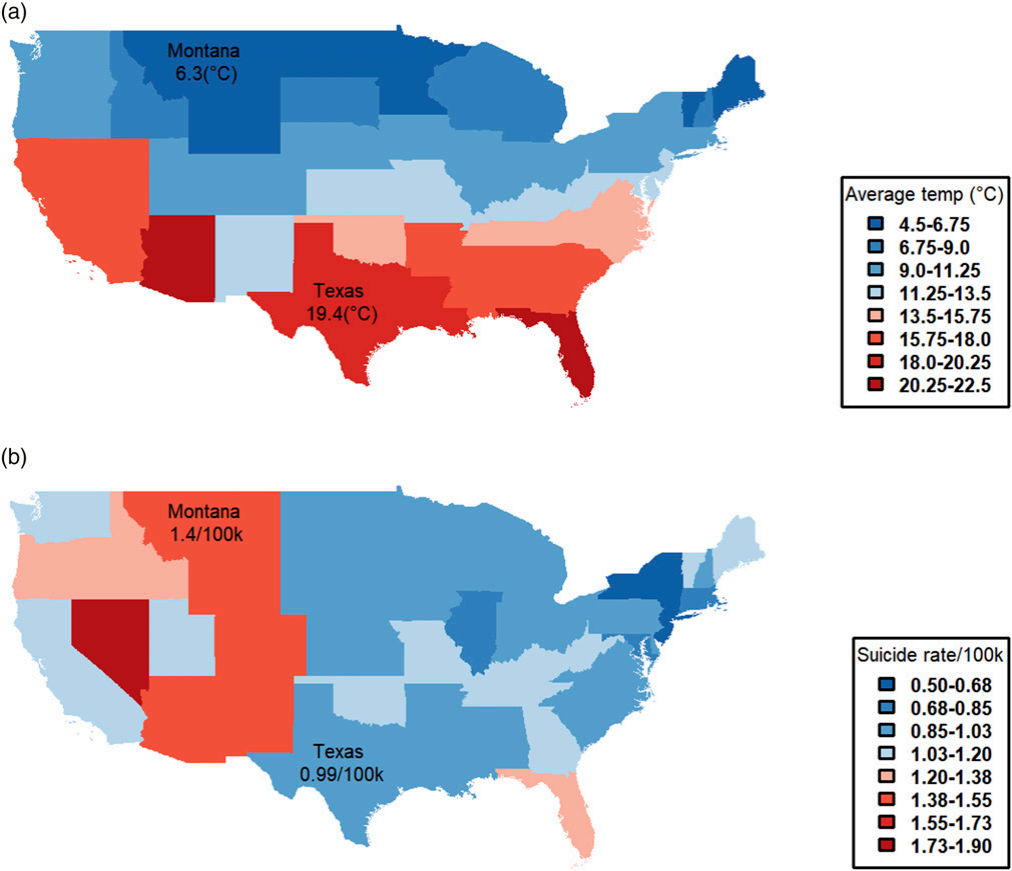
US average temperature and suicide rate per 100k population by state.

Figure 1 juxtaposes the relationships between temperature and suicide estimated by aggregated (Figure 1(a)) and disaggregated data (Figure 1(b)). Figures 2(a) and (b) show the distribution of US state temperatures and suicide rates averaged over the period 1968–2004, respectively.

Table 3 summarises the information contained in the other tables and figures.

**Table 3.** Methodological features: descriptions and interpretations

Feature	Description	Interpretation
Table 1	Provides a summary of articles cited as authority for a relationship between climate change and suicide by Burke et al.^7^	
Table 2	Outlines the features of a set of linear regressions estimating relationships between temperature and suicide. The table summarises information about regressions performed on the same data set using the three different statistical models formally described in the Method section and below. The Coefficient column shows the size and direction of the relationship between predictors and outcomes; and Adjusted *R*^2^ describes how much of the variance in the outcomes is explained by the predictors (with a higher score indicating more variance is explained)	
• Burke et al. model	The model used by Burke et al.^7^ in the original paper analysed data at the suburb level and predicted the suicide rate for each suburb based on how much the temperature for that suburb in each calendar month (eg Jan, Feb and Mar) differed from the average temperature for that suburb for that calendar month across all years	The Burke et al. Model found no relationship between rain and suicide; it reported an increase in suicide in suburbs in calendar months that had higher temperatures than the average for that suburb and calendar month across all years; that is, the average suicide rate was expected to be higher for a suburb in Jan 2022 if the temperature was higher than the average for that suburb across all Januaries in the period studied
• Aggregate-year model	This model aggregated the suicide and temperature data by year, and estimated the national yearly suicide rate predicted by temperature for that year	The Aggregate-year model reported a significant relationship between increased annual temperature and decreased suicide rate for that year
• Aggregate-state model	This model aggregated the suicide rate and temperature data by state, and estimated the suicide rate predicted by state across all years	The Aggregate-state model reported a significant relationship between an increased average state temperature and an increased suicide rate for thatstate
Figure 1(a)	Plots the relationships between temperature and suicide in the US described by the Aggregate-year and Aggregate-state models. Modelled on Burke et al.^7^ the figure describes the % change in suicide rate predicted by changes in average yearly temperature relative to the average temperature across all years	The solid blue line shows that when aggregated by year, an increase in average US temperature is associated with a decrease in suicide rate
		The dotted blue line shows that when aggregated by US state, an increase in temperature is associated with an increase in suicide rate
Figure 1(b)	Plots the relationships between temperature and suicide in the US described by the Burke et al.^7^ model	Burke et al.^7^ reported multiple variations of their model, all of which showed that there was a higher suicide rate for individual suburbs in months that had relatively higher temperatures than the average temperature for that month across all years; for example, a suburb with a higher temperature in Jan 2022 than the average for that suburb across all Januaries, would have a higher suicide rate
	This plot was included to provide a baseline against which to interpret Figure 1(a)	
Figure 2(a)	Plots the average temperature for each US state across the entire time period, with more blue indicating lower temperature and more red indicating higher temperature	Shows the expected pattern that the northern US states were, on average, colder than the southern US states across the years of the study
		Montana and Texas are specifically identified to provide a comparison
Figure 2(b)	Plots the average suicide rate per 100k by US state where blue indicates a lower and red a higher suicide rate	Shows a relatively high rate of suicide in western US states excluding California and Washington
		Montana and Texas are specifically identified to provide a comparison

## Discussion

These results demonstrate that the research cited to suggest an association between climate change and increased suicide^3^ does no such thing. The review cited by the RCPsych^5^ only examines periodic variations of the seasonality hypothesis, that suicides are unevenly distributed across time periods. The reanalyses show that monthly associations between increased temperature and suicides co-exist with associations between increased temperature and decreased suicides across years.

This paradox reflects the fact that studies which examine how periodic variations in temperature across short periods of time influence suicide cannot test whether temperatures increased by climate change increase suicide rates. Burke et al.^8^ reported that individual suburbs report above average suicide rates during calendar months with temperatures above the average for that suburb for that calendar month. For example, if the temperature of suburb A in January 2022 was higher than the average temperature of suburb A across all Januaries in the period being considered, suicide would be expected to be higher for January 2022 than the average of all other Januaries for suburb A. The unstated corollary is that suicide would be expected to be lower in months with lower than average temperatures. As a result, increases in average temperature caused by climate change would be expected to have no impact on suicide rate, because it would not affect the number of months with above- and months with below-average temperature.

Figures 1 and 2 demonstrate that periodic variations of the seasonality hypothesis provide no evidence on the impact of climate change on suicide. For climate change to have an impact on suicide rates there would have to be a relationship between temperature change sustained across decades and extending across large geographic areas, not across suburbs and months. Figure 1(a) shows that the relationship between temperature and suicide in the US aggregated by years is negative (Year.aggregate line); and Figure 2 shows that there is a completely different distribution of US states with high average temperatures and high suicide rates. Any claim that a sustained increase in temperature causes a sustained increase in suicide risk would have to explain why Texas, with amongst the highest average temperature of US states, has a below average rate of suicide, while Montana shows the opposite pattern.

While it is true that simple linear regressions do not exclude confounding factors, it is equally true that they provide the most direct description of correlations in observed data. If it is assumed that confounds drive the association between higher average US temperature and lower suicide, the aggregated regressions demonstrate that the confounds overwhelm any putative effect of increased temperature due to climate change. That is, even if it is assumed without evidence that increased temperatures due to climate change increase suicide rates, that relationship is nowhere near strong enough to influence the observed negative relationship at the national level between 1968 and 2004 in the US.

This analysis lends weight to the common-sense view that the priority of health and mental health systems should be high quality health care, not climate change action. Reanalyses of the Burke et al.^8^ data indicate that even if it is assumed that increased temperature due to climate change increases the risk of suicide, other factors with much bigger influence should be the focus of intervention. Climate change action appears unlikely to have a measurable impact on suicide, and it is unknown whether adding climate change goals to the priorities of health systems will impact patient care. The recent reversal of aggressive expansion of gender-affirming care based on little more than good intentions demonstrates the dangers to patients, health systems, and trust in institutions of changing health practices in advance of evidence.^10^

The Thompson et al.^6^ and Burke et al. papers^8^ illustrate common techniques for misrepresentation of scientific evidence. The first is uncomplicated misrepresentation of scientific results as providing evidence for the impact of climate change when they do not. Thompson et al.^6^ acknowledge this in their discussion: ‘None of the included studies [in our systematic review] looked specifically at climate change, whereas the findings of our review support the assertion that the risk of suicide and other mental health outcomes is likely to increase in line with climate projections’.

Second is to combine individually unconvincing studies to make claims about the importance of the larger set. The 17 of 35 articles on suicide reviewed by Thompson et al.^6^ are combined with multiple other study types. This agglomeration of disparate studies distracts attention from the fact that the suicide studies are not designed to consider climate change, and from the fact that none of the other studies establish strong evidence for a relationship between climate change and mental health outcomes either.

The RACP review endorsed by the RANZCP^4^ collects a jumble of studies alongside a narrative review of policy and institutional literature and a set of case studies. Most relevant for the current analysis, the RACP review includes an economic analysis of bushfires, without providing any evidence that climate change is related to bushfires, or that climate change action will decrease the risk or impact of bushfires. There is no scientific rationale for the inclusion of this economic analysis in the review, which suggests it has been included as an appeal to emotion.

The final technique is to ignore the evidence provided when making recommendations. The RACP review,^4^ for example, recommends that Australian governments commit to net zero healthcare by 2040 despite having provided little evidence of the relationship between climate change and healthcare; no evidence that net zero healthcare is possible; and no evidence of the impact of net zero healthcare on patient outcomes or health system sustainability.

## Limitations

The present study critically analyses the evidence for assertions and recommendations predicated upon a link between climate change and mental health. The study does not attempt to establish there is no relationship between climate change and mental health outcomes, only that the cited evidence does not establish a relationship. The novel linear regressions employed rely upon the data provided under an open licence and do not attempt to correct for confounds. This is an explicit choice in order to demonstrate problems with the original analyses and conclusions.

## Conclusions

Climate change advocates are passionate about their cause, and this appears to be associated with biases in their presentation of scientific evidence. The present study suggests that policy recommendations based on the climate change literature require a high bar of critical analysis to avoid acting on misrepresentations of the evidence base. None of the literature identified by the RANZCP demonstrates a relationship between climate change and suicide, and indeed, none has a design capable of demonstrating such a relationship. The recommendation to deliver net zero healthcare by 2040 in the absence of any evidence about how to achieve this at a systems level, whether it is desirable, and what the costs would be, is consistent with faith that climate change action is morally justifiable, and not with scientific evidence that it is practically desirable, or even possible.
